# Barriers to Diabetes Self-Management and Family Support Among Older Adults in Resource-Limited Settings in Nigeria

**DOI:** 10.1093/geroni/igaf122.847

**Published:** 2025-12-31

**Authors:** Jimmy Reyes

**Affiliations:** Proteus Clinic, Des Moines, Iowa, United States

## Abstract

Diabetes is a growing public health concern among older adults, particularly in resource-limited settings where access to healthcare is constrained. This study examined diabetes self-management and family support among older adults in Adamawa State, Nigeria, using a cross-sectional, descriptive design with 810 participants across five local government areas. Data were collected through validated surveys and collected using the Kobo Toolbox. Results indicated that older adults face significant barriers to diabetes management, including lack of health insurance (89%), high out-of-pocket expenses, and limited access to medication, with 74.5% of rural participants not taking Metformin. Dietary and lifestyle interventions remain insufficient, as only 21% of participants reported receiving guidance on low-fat diets, and 34% engaged in regular physical activity. Correlation analyses revealed significant associations between diabetes management and factors such as health insurance status (p < .001), adherence to prescribed medication (p < .001), and healthcare follow-up (p < .001). Regression analysis identified local government area, diabetes diagnosis duration, and healthcare access as key predictors of diabetes self-management (R² = 0.221, p < .001). The findings underscore the need for targeted interventions to improve diabetes care among older adults, particularly in rural communities. Strengthening healthcare access, financial support mechanisms, and culturally tailored education programs could enhance self-management and reduce diabetes-related complications in this vulnerable population.

